# Prognostic value of the video head impulse test in sudden sensorineural hearing loss with vertigo: a systematic review and meta-analysis

**DOI:** 10.3389/fneur.2025.1756795

**Published:** 2026-01-12

**Authors:** Qiang Guo, Ying Lin, Zhilin Wang, Pengfei Hang, Dingjun Zha

**Affiliations:** Department of Otolaryngology, Head and Neck Surgery, Xijing Hospital, PLA Air Force Medical University, Xi’an, China

**Keywords:** audiological prognosis, hearing recovery, sudden sensorineural hearing loss, vestibular test, video head impulse test

## Abstract

**Objectives:**

To characterise the pattern of video head impulse test (vHIT) impairments in patients with sudden sensorineural hearing loss with vertigo (SSNHL-V), and to assess its correlation with audiological prognosis.

**Design:**

We systematically searched four databases (PubMed, Embase, Scopus, Cochrane Library) from inception to February 24, 2025, performed meta-analysis using Stata 18, and had the protocol prospectively registered with PROSPERO (CRD420251025900).

**Results:**

Among patients with SSNHL-V, impairment of the posterior semicircular canal (PSCC) was found to be more common than that of the horizontal semicircular canal (HSCC) or anterior semicircular canal (ASCC). The pooled prevalence of PSCC abnormality was 50% (95% CI: 0.40–0.60; *I*^2^ = 87.65%, *z* = 14, *p* < 0.05). Regarding auditory prognosis, patients with reduced PSCC gain on vHIT had a 4.14-fold increased risk of poor hearing recovery (95% CI: 2.64–6.51; *I*^2^ = 7%, *p* = 0.36; *z* = 6.17, *p* < 0.05). Similarly, reduced HSCC gain was associated with a 3.06-fold increased risk (95% CI, 1.72–5.44; *I*^2^ = 0%, *p* = 0.96; *z* = 3.82, *p* < 0.05).

**Conclusion:**

In patients with SSNHL-V, vHIT assessment revealed a higher rate of PSCC dysfunction compared to other semicircular canals, and impaired PSCC function serves as a significant predictor of auditory prognosis.

## 1 Introduction

In the late 19th and early 20th centuries, research into the relationship between head and ocular movements revealed that any rotation of the head induces a unique activation pattern across the six semicircular canals of the vestibular system (1), laying the groundwork for the study of the vestibulo-ocular reflex (VOR). Upon activation of the receptor of the semicircular canals by head rotation, neurons drive ocular movements via rapid neural pathways to correct for head motion. This ensures ocular movements match head movements in angular velocity while opposing them in direction, thereby stabilizing retinal imaging and maintaining visual clarity. In clinical audiological testing, calibrated audiometers/headphones are used to deliver precisely controlled acoustic stimuli and to measure and record patient responses. Similarly, in clinical VOR assessment, tests such as the head-shaking nystagmus test (HSNT) and the head impulse test (HIT) employ natural head acceleration as the stimulus. However, unlike the calibrated audiometer, this mode of stimulation is difficult to control with precision in a clinical setting. In contrast, the vHIT enables the precise measurement of the ocular movement response corresponding to each head impulse. According to MacDougall et al., the scleral search coil method, which is regarded as the gold standard for vestibular-ocular testing in laboratories, and vHIT have similar diagnostic accuracy (2–4).

Sudden sensorineural hearing loss (SSNHL) is clinically characterized by a loss of over 30 dB HL in at least three contiguous frequencies occurring within 72 h (5). Common complications of SSNHL include tinnitus, ear fullness, vertigo, and unsteadiness. The phenomenon of vestibular involvement in this condition was first reported in 1949 (6). Given the embryological and anatomical interrelationship between the cochlear and vestibular systems, approximately 30–60% (7–9) of patients with SSNHL experience vestibular dysfunction concurrently with their hearing impairment. While vestibular dysfunction has been identified in multiple studies as a key indicator of hearing prognosis, there is no consensus on which specific test holds superior predictive value. All six semicircular canals’ high-frequency VOR function may be individually evaluated using the vHIT, which allows for both qualitative and quantitative analysis of the semicircular canals and their neural pathways. Gain and the pathological catch-up saccades are two of the main criteria of vHIT that indicate the state of vestibular compensation and the functional status of the vestibular system. Compared to other vestibular function tests, vHIT offers better patient tolerance and can be conveniently administered as a bedside examination, leading to its widespread application and in-depth study across various vestibular disorders. This systematic review examines the performance of the vHIT in assessing patients with SSNHL-V and explores the correlation between vHIT findings and audiological prognosis.

## 2 Materials and methods

### 2.1 Data sources and search strategy

This systematic review was conducted in accordance with the PRISMA guidelines (10). Two authors independently performed a comprehensive literature search of the PubMed, Cochrane Library, Embase, and Scopus databases for studies published up to February 24, 2025. No restrictions were placed on the publication date. Using all available free-text synonyms and the pertinent Medical Subject Headings (MeSH), such as “Hearing Loss, Sensorineural” and “video head impulse test”, the literature search was conducted. The complete search strategy for all databases is available in Supplementary Table S1. The following were the requirements for inclusion in the study: (1) the study population had to be SSNHL-V patients; (2) vHIT results included in the vestibular assessment; and (3) availability of pure-tone audiometry results or data on hearing recovery. Exclusion criteria included: (1) non-English publications; (2) animal studies, reviews, conference reports, and case reports; and (3) studies with incomplete data necessary for analysis.

### 2.2 Data abstraction and quality assessment

In accordance with the guidelines, the retrieved potential studies were imported into the reference management software EndNote 21. Two staff members independently conducted a preliminary assessment and exclusion based on titles and abstracts. The information and data used to determine the inclusion of studies were independently extracted from each selected study. The hearing recovery rate was defined as the proportion of patients with incomplete hearing recovery in each group relative to the total number of patients in that group. The retrieved characteristics included: the surname of the primary author, year of publication, country, research design, study duration, number of SSNHL subjects, abnormal rate of vHIT results, audiologic test outcomes, and hearing recovery.

The quality of the included studies was evaluated separately by two authors using the Newcastle-Ottawa Scale (NOS). The quality was examined across three dimensions: study population, outcome assessment, and comparability of groups, comprising a total of 8 items. Each item was scored using a star system, with the overall score ranging from 0 to 9 stars. A superior score signifies enhanced study quality. Studies having a score of 6 or above were deemed high-quality (11). The quality assessment results are presented in Supplementary Table S2.

### 2.3 Statistical analysis

STATA 18 software was used to conduct the meta-analysis, and forest plots were used to display the findings. The pooled abnormal rate of the semicircular canal in vHIT results was determined through a single-arm meta-analysis. The hearing recovery rate was coded as dichotomous data, and the relationship between auditory prognosis and vHIT was analyzed by combining odds ratios (ORs) using the Mantel–Haenszel method. The *Q*-test and *I*^2^ test were utilized to quantify heterogeneity across the included studies. A *p*-value < 0.05 and/or *I*^2^ > 50% indicated substantial heterogeneity (12). A fixed-effects model was employed when *I*^2^ < 50% and *p* > 0.1; otherwise, a random-effects model was utilized. The assessment of publication bias in the included studies was conducted using funnel plots and symmetry tests.

## 3 Results

### 3.1 Literature search results

**Figure 1:**
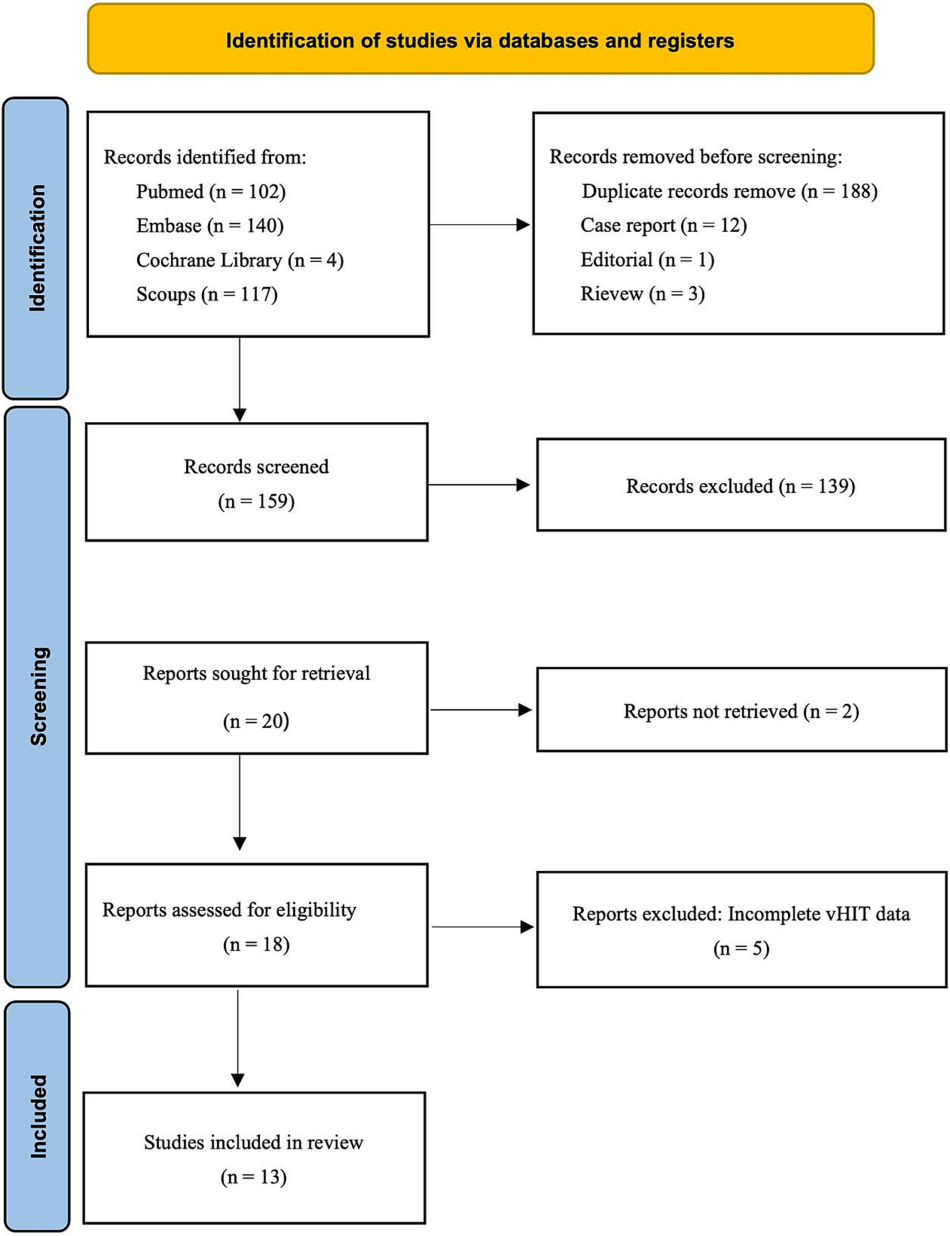
The PRISMA flowchart of selecting the relevant studies for this review is depicted.

The PRISMA flowchart in Figure 1 illustrates the process of searching, deleting, and filtering for relevant studies. As of February 24, 2025, a total of 363 records were identified through searches using subject headings and free-text terms for “Hearing Loss, Sensorineural” and “video head impulse test”. Before abstract screening, 188 duplicate records were removed. Among the remaining records, 12 case reports, 3 review articles, and 1 editorial were excluded. After screening titles and abstracts, 139 records were further excluded. Two studies could not be retrieved in full text (13, 14), and five studies did not provide detailed vHIT parameter data (15–18). Ultimately, 13 studies were deemed eligible for inclusion (19–31).

### 3.2 Study characteristics

Table 1 presents the essential characteristics of the 13 selected studies, conducted between 2016 and 2025 and covering China, Australia, North Korea, South Korea, and Japan. These studies included 1,141 participants, with sample sizes ranging from 23 to 148, and a male-to-female ratio of 501:640. Four studies included other patients with acute vestibular syndrome, such as vestibular neuritis (VN) and Ramsay Hunt syndrome (RHSD), to compare vHIT results with those of patients with SSNHL-V (19, 21, 22, 24). The criteria for hearing recovery varied across the included studies. Two studies employed Siegel’s criteria (25, 29), two defined recovery as a pure-tone average (PTA) within 10 dB HL of the unaffected ear (23, 26), one applied a threshold of PTA < 20 dB HL (20), and another used PTA < 25 dB HL as the criterion (31). The median Newcastle-Ottawa score for the 13 included studies was 4 (range: 4–6; See Supplementary Table S2). All studies were observational studies with moderately low study quality.

**Table 1:** Literature reports of selected studies.

Study	Research type	Patients with SSNHL-V	Patients with SSNHL without vertigo	vHIT results			Hearing recovery criteria
				HSCC	ASCC	PSCC	
Liu Y (2025), (19)	R	70	0	16/70	3/70	39/70	
Qian Y (2024), (20)	R	112	114	24/112	20/112	60/112	PTA < 20dBHL
Nakamichi N (2024), (21)	R	15	0	8/15	2/15	11/15	
Liu Y (2023) (22)	R	57	0	12/57	3/57	30/57	
Hong JP (2023), (23)	R	73	79	15/73	4/73	41/73	Within 10 dB HL of unaffected ear
Hong JP (2023), (24)	R	81	0	33/81	9/81	42/81	
Hao W (2023), (25)	P	86	0	46/86	10/86	48/86	Siegel
Cho JW (2023), (31)	R	23	0	16/23	11/23	19/23	PTA < 25 dB HL
Seo HW (2022), (26)	R	54	81	8/54	7/54	20/54	Within 10 dB HL of unaffected ear
Jiang Z (2021), (27)	R	29	21	2/29	10/29	17/29	
Lee JY (2020), (28)	R	71	0	10/71	5/71	21/71	
Byun H (2020), (29)	R	148	0	25/148	19/148	28/148	Siegel
Pogson JM (2016), (30)	R	27	0	11/27	8/27	20/27	

R, retrospective; P, prospective.

**Figure 2:**
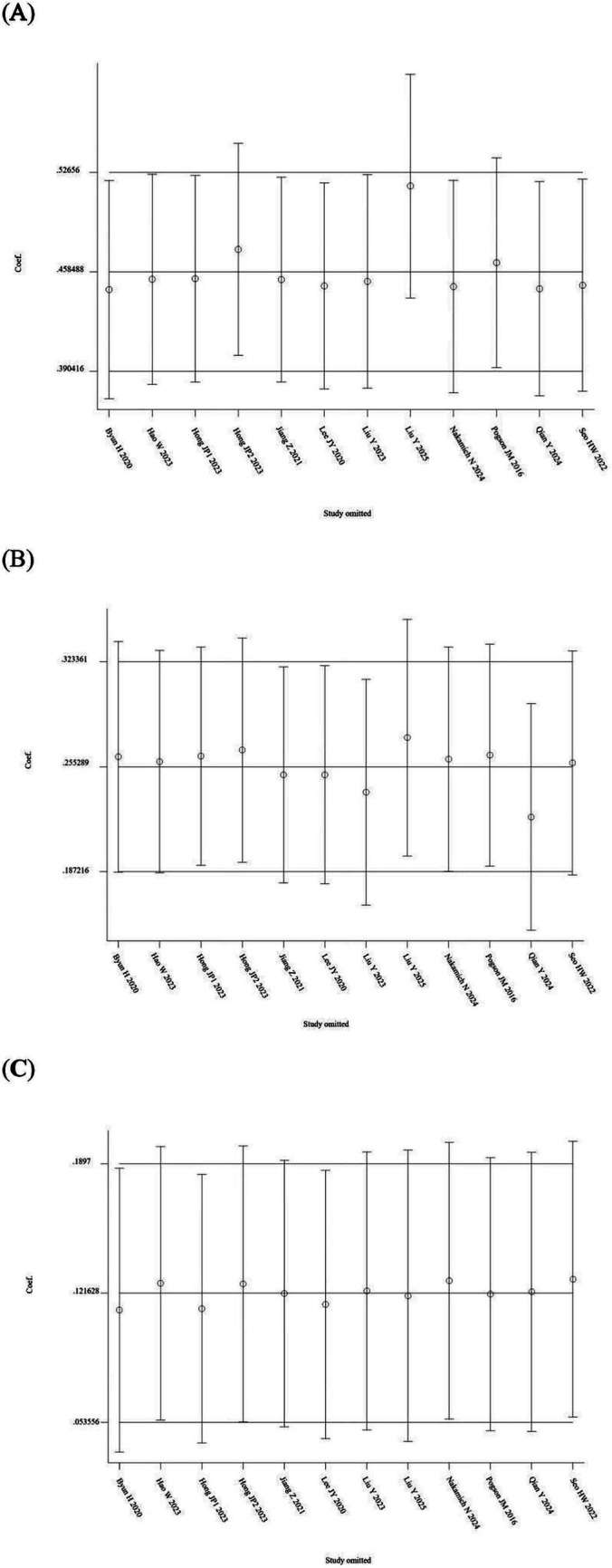
Sensitivity analysis of the random-effects model of the three semicircular canal abnormality rates. **(A)** Sensitivity analysis of PSCC. **(B)** Sensitivity analysis of HSCC. **(C)** Sensitivity analysis of ASCC.

**Figure 3:**
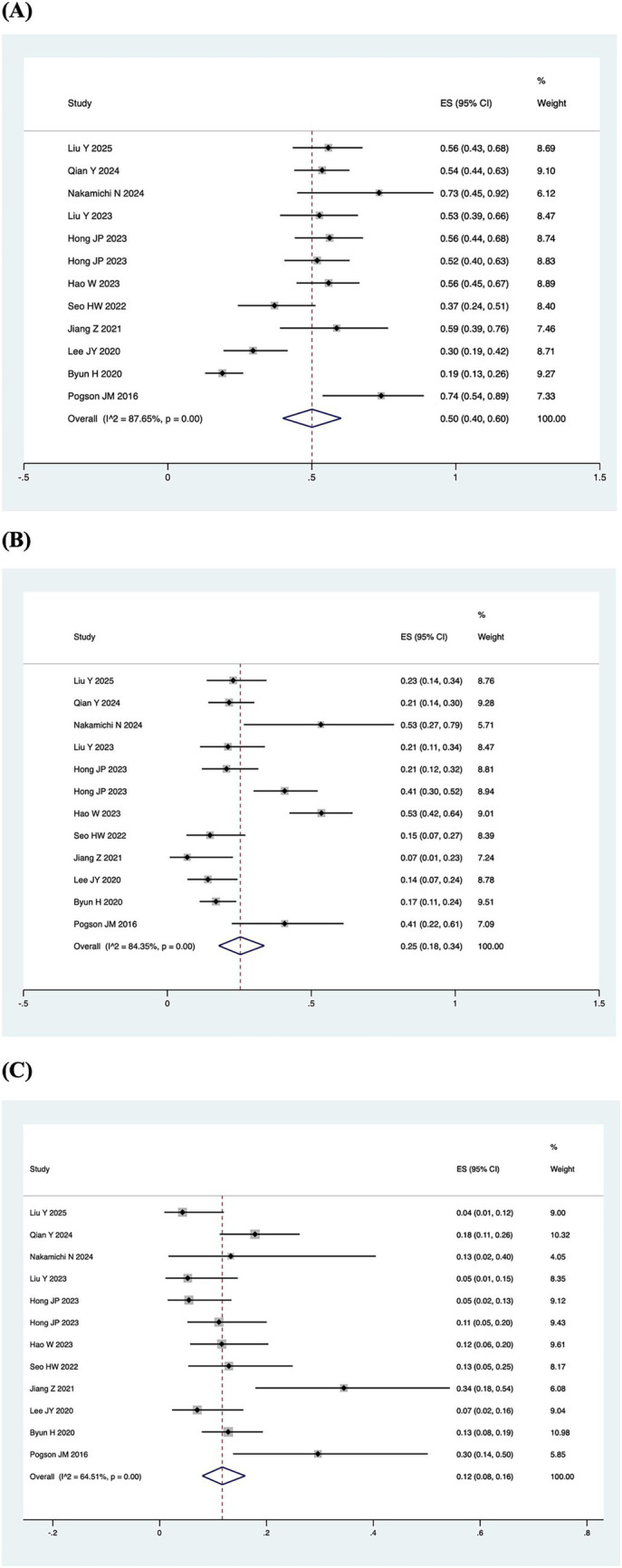
Pooled abnormal rate of the semicircular canal in the vHIT result. **(A)** Forest plot diagram of the abnormality rate in PSCC. **(B)** Forest plot diagram of the abnormality rate in HSCC. **(C)** Forest plot diagram of the abnormality rate in ASCC.

**Figure 4:**
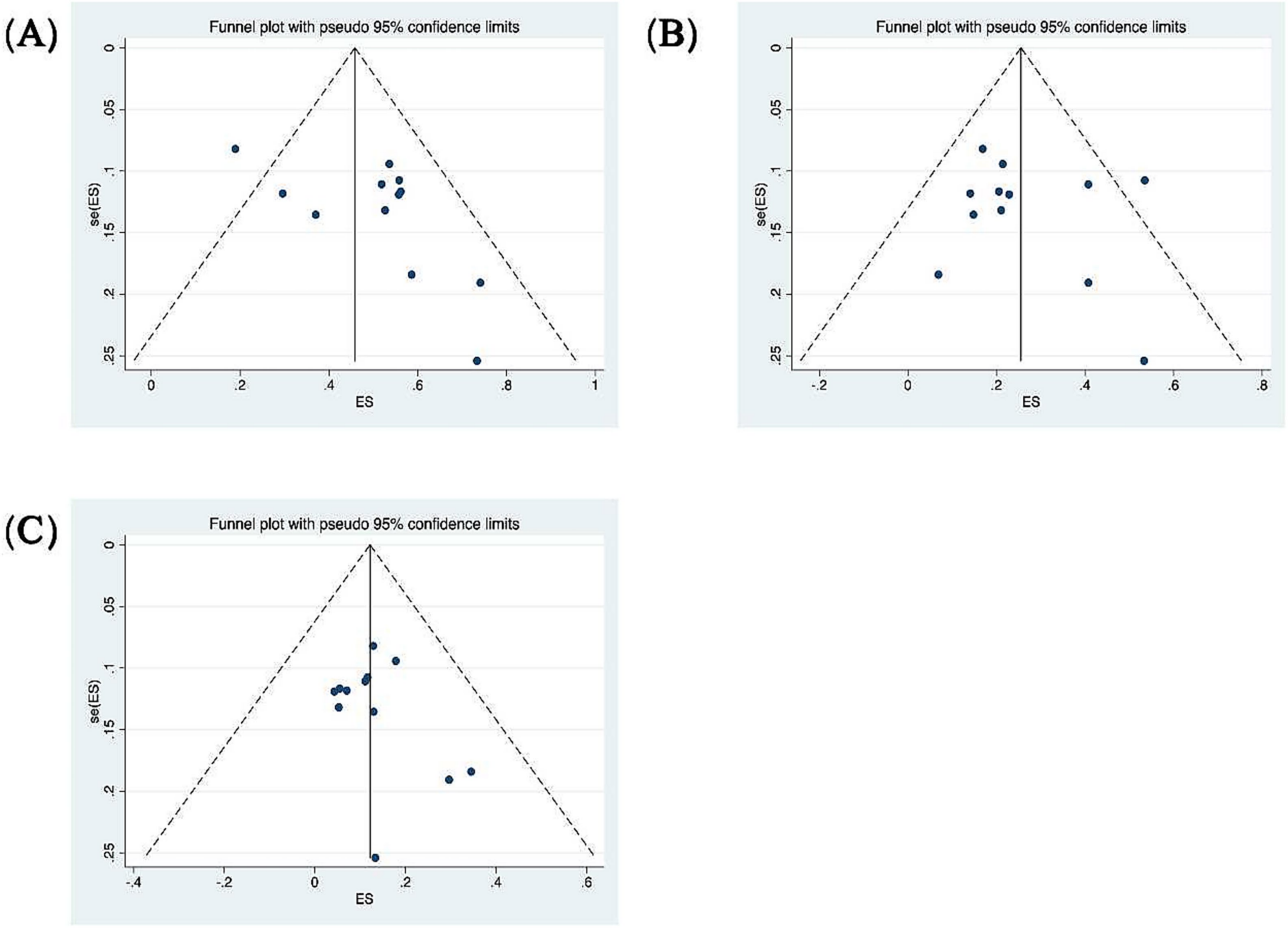
Funnel plots for the evaluation of publication bias. **(A)** Funnel plot of the included studies in the meta-analysis of the abnormality rate in PSCC. *p* = 0.373 > 0.05, funnel plot symmetry, no publication bias. **(B)** Funnel plot of the included studies in the meta-analysis of the abnormality rate in HSCC. *p* = 0.732 > 0.05, funnel plot symmetry, no publication bias. **(C)** Funnel plot of the included studies in the meta-analysis of the abnormality rate in ASCC. *p* = 0.945 > 0.05, funnel plot symmetry, no publication bias.

A total of 7 out of the 13 included studies demonstrated a higher prevalence of abnormal vHIT results for the PSCC relative to the other canals in patients with SSNHL-V (19–24, 28). One study (Jiang et al., 2021) was excluded from this subgroup owing to insufficient data on canal-specific abnormality rates (27). Heterogeneity testing across the remaining 12 studies revealed significant heterogeneity, with *I*^2^ > 50% and *p* < 0.1 of the *Q*-test. A sensitivity analysis was conducted on 12 studies (Figure 2), revealing that no single study significantly disrupted the meta-analysis results, indicating robust stability of the research. Consequently, a random-effects model was utilized to pool the abnormal rates of the three semicircular canals according to vHIT results. The analysis showed an abnormal PSCC rate of 50% (95% CI: 0.40–0.60; *I*^2^ = 87.65%, *p* = 0; *z* = 14, *p* < 0.05), an abnormal HSCC rate of 25% (95% CI: 0.18–0.34; *I*^2^ = 84.35%, *p* = 0; *z* = 10.15, *p* < 0.05), and an abnormal ASCC rate of 12% (95% CI: 0.08–0.16; *I*^2^ = 64.51%, *p* = 0; *z* = 9.5, *p* < 0.05) (Figure 3). The assessment of publication bias was conducted through a funnel plot (Figure 4), with symmetry tests revealing no significant evidence of publication bias.

### 3.3 Hearing recovery based on vHIT results

**Figure 5:**
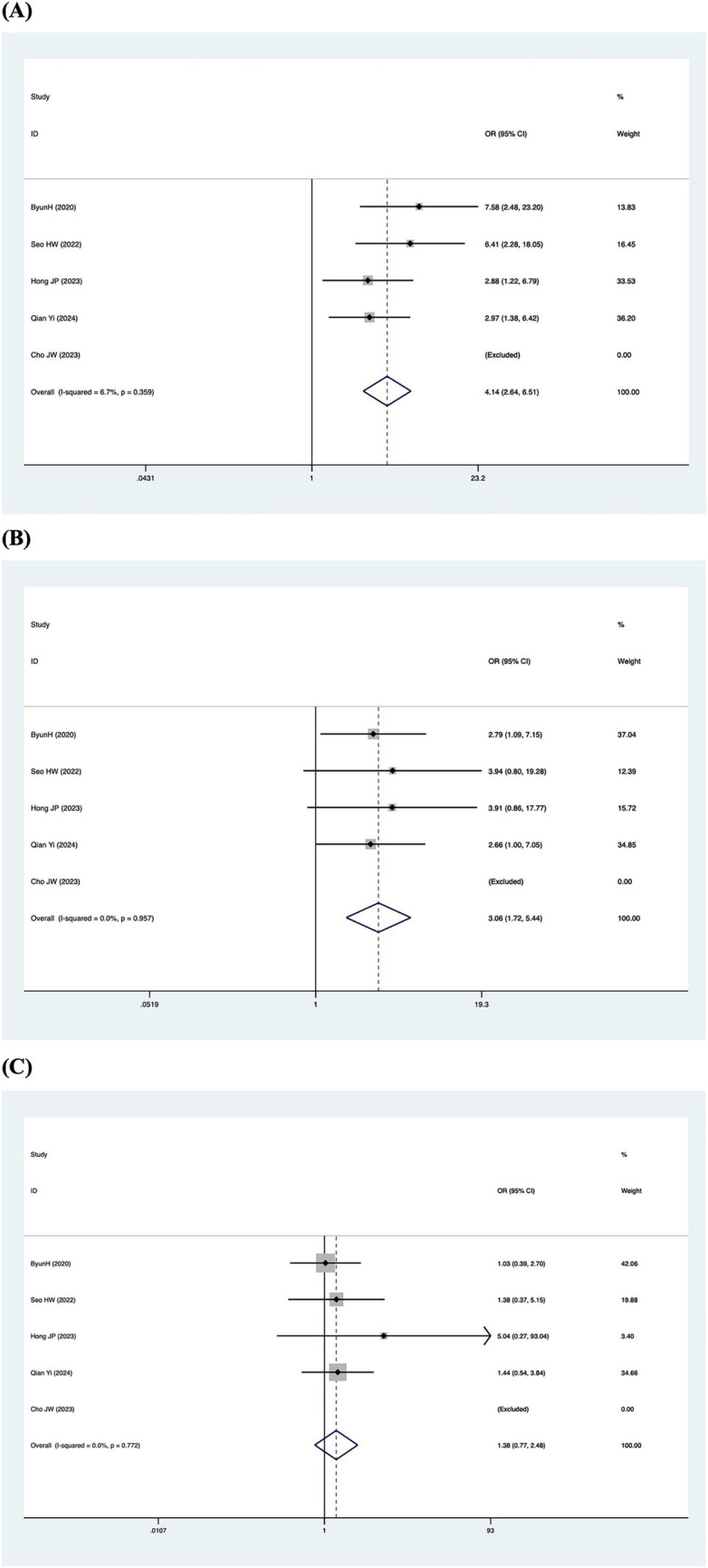
Forest plots of the synthesized data from the selected studies. **(A)** Forest plot diagram of hearing recovery and PSCC results. **(B)** Forest plot diagram of hearing recovery and HSCC results. **(C)** Forest plot diagram of hearing recovery and ASCC results.

**Figure 6:**
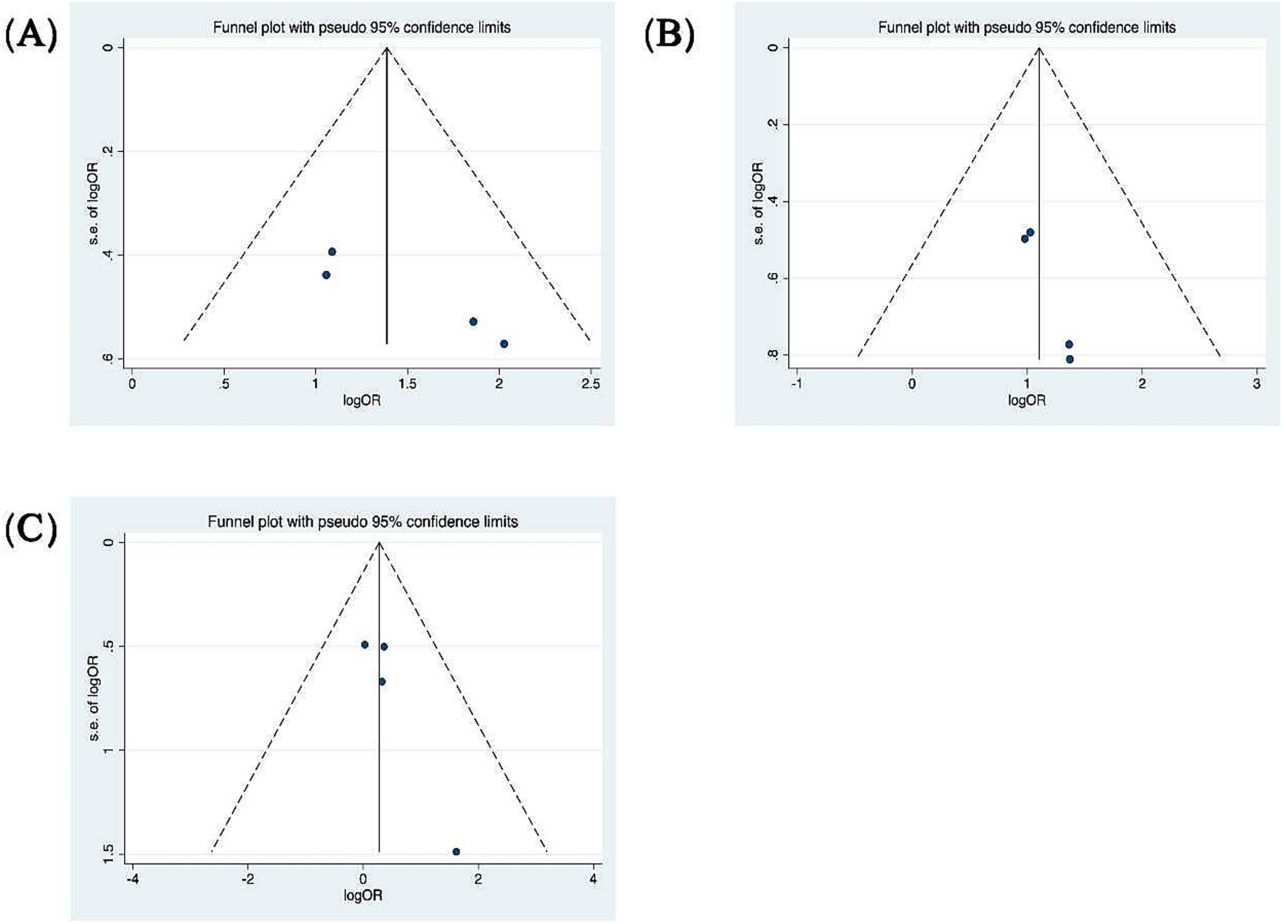
Funnel plots for the evaluation of publication bias. **(A)** Funnel plot of the included studies in the meta-analysis of hearing retrieval and PSCC. *p* = 0.045 > 0.05. **(B)** Funnel plot of the included studies in the meta-analysis of hearing retrieval and HSCC. *p* = 0.017 < 0.05. **(C)** Funnel plot of the included studies in the meta-analysis of hearing retrieval and ASCC. *p* = 0.111 > 0.05.

Patients were categorized according to whether the gain values for each semicircular canal (abnormal gain: <0.8 for the HSCC, <0.7 for the vertical semicircular canals) in the vHIT results were normal or not, and the association with inadequate hearing recovery was examined using a dichotomous method. Heterogeneity testing across the five included studies revealed *I*^2^ < 50% and a *Q*-test *p*-value > 0.1, indicating no significant heterogeneity among the studies (20, 23, 26, 29, 31). A fixed-effects model was utilized to assess the subgroup outcomes. The weighted mean odds ratio was 3.06 for the HSCC (95% CI: 1.72–5.44; *I*^2^ = 0%, *p* = 0.96; *z* = 3.82, *p* < 0.05), 1.38 for the ASCC (95% CI: 0.77–2.48; *I*^2^ = 0%, *p* = 0.77; *z* = 1.07, *p* = 0.29), and 4.14 for the PSCC (95% CI: 2.64–6.51; *I*^2^ = 7%, *p* = 0.36; *z* = 6.17, *p* < 0.05). These results indicate a statistically significant association between reduced PSCC gain and poor auditory prognosis (Figure 5). Publication bias was assessed using a funnel plot (Figure 6), and symmetry testing confirmed the funnel plot was symmetric, suggesting no significant publication bias.

## 4 Discussion

Numerous studies have established that vertigo serves as a negative prognostic factor for hearing recovery. However, there is no consensus regarding which specific vestibular function test most effectively predicts hearing outcomes. vHIT is a rapid, easily executable, and well-tolerated examination that can quantitatively assess high-frequency VOR function (4). The main aim of this systematic review and meta-analysis is to examine the impairment patterns of vHIT and the relationship between vHIT outcomes and hearing prognosis in patients with SSNHL-V.

Among the 13 studies ultimately included, four compared the vHIT impairment patterns between patients with other acute vestibular syndromes (VN and RHSD) and those with SSNHL-V (19, 21, 22, 24). The comparative analysis indicated that patients with RHSD and VN exhibit significantly reduced VOR gain and an increased occurrence of pathological saccades in the HSCC and ASCC relative to the SSNHL-V group (19). In cases of RHSD, the impairment in gain is more severe, with a broader involvement of impaired semicircular canals, often manifesting as combined damage to all three semicircular canals (24). Due to the neural clamping mechanism involved in vestibular compensation, not only is the affected side compromised, but the healthy side is also impacted, exhibiting a reduction in gain over a certain period (32). In contrast, among VN patients, the HSCC was the most frequently affected, followed by the ASCC and PSCC (19, 21, 22). This pattern of higher involvement rates for the HSCC and ASCC compared to the PSCC was also corroborated in studies of VN patients by Magliulo and Taylor, respectively (33, 34).

vHIT exhibits distinct impairment patterns that may be associated with viral infection characteristics. RHSD is a polycranial neuropathy triggered by the reactivation of the varicella-zoster virus (VZV) latent in cranial nerve ganglia (35). Due to the neurotropic and polyneuropathic nature of VZV, the lesion scope in RHSD often extends beyond a single nerve. Combined involvement of the 7th (facial) and 8th (vestibulo-cochlear) cranial nerves is most common, and it may also affect the 5th, 9th, and 10th cranial nerves (36). In rare cases, inflammation may retrogradely extend to the brainstem, resulting in vestibular pathology exhibiting both peripheral and central features (37). Consequently, vHIT reveals a more extensive (involving all semicircular canals) and severe (more significant gain reduction) impairment pattern. Although the exact etiology of vestibular neuritis (VN) remains unclear, it is generally attributed to viral infections (particularly HSV-1) (38). The lesion in VN is primarily confined to the vestibular nerve itself, aligning more closely with its anatomical distribution and demonstrating greater selectivity. The predominant vHIT findings in VN are isolated impairment of the HSCC or a combination of impairments in both the HSCC and ASCC.

Compared to the two aforementioned acute vestibular syndromes, patients with SSNHL-V exhibit a higher impairment rate of the PSCC. vHIT testing in SSNHL patients reveals that the PSCC has the highest impairment rate, followed by the HSCC, while the ASCC shows the lowest rate. Common impairment patterns manifest as either isolated or combined PSCC dysfunction. Furthermore, studies have found that PSCC dysfunction has a greater impact on poor hearing prognosis, with reduced PSCC gain increasing the likelihood of poor hearing recovery by 4.14-fold. The explanation for this phenomenon is primarily supported by the inference of vascular compromise. The HSCC, ASCC, the utricular macula, and the superior part of the saccular macula receive blood supply from the anterior vestibular artery (39). As a branch of the common cochlear artery, the posterior vestibular artery supplies the PSCC and the part of the saccular macula. The lack of collateral circulation makes these structures more vulnerable to vascular impairment. Research involving animals has demonstrated that ischemia persisting for 30 min or more can result in irreversible damage (40). In another meta-analysis investigating cVEMP (cervical Vestibular Evoked Myogenic Potential) in SSNHL patients, abnormal cVEMP findings were associated with a 3.22-fold increased risk of poor hearing prognosis (41). This finding is consistent with our conclusion, indicating that involvement of the inferior vestibular pathway has a strong influence on poor hearing prognosis.

In a case of left-sided SSNHL-V, inner ear MRI 3D-FIESTA sequences revealed a filling defect in the PSCC, indicative of fibrosis due to ischemia, which further supports the vascular theory (42). Furthermore, a multivariate analysis by Byun et al. indicated that PSCC involvement exhibited the highest odds ratio among the considered clinical factors (e.g., age, initial hearing level) (29). While many unaccounted variables may influence auditory recovery in SSNHL, the relationship between partial/absent recovery and vascular pathophysiology should not be regarded as coincidental.

## 5 Limitations

This study presents several limitations. First, the included studies are limited in number and do not consist of multicenter studies or controlled trials. Second, all incorporated studies were retrospective in design, which may introduce significant confounding factors. Third, nearly all the studies employed different assessment methods or combinations of tests to evaluate vestibular function, making it difficult to compare the results. Currently, a standardised, multi-center-validated prognostic scoring system for hearing outcomes based on vestibular function test parameters has not yet been established.

## 6 Conclusion

This meta-analysis indicates that in patients with SSNHL-V, the rate of impairment is higher in the PSCC than in the other semicircular canals. The findings suggest that reduced vHIT gain of either the HSCC or PSCC is associated with an increased risk of poor hearing prognosis. Notably, PSCC involvement carries a substantially greater risk, with 4.14-fold higher odds of poor hearing recovery, establishing it as a key predictor. Furthermore, standardised vHIT assessment shows potential clinical utility for informing treatment decisions, predicting audiological outcomes, and guiding vestibular rehabilitation in all SSNHL patients with pathological vHIT, regardless of the presence of subjective vertigo.

## Data Availability

The raw data supporting the conclusions of this article will be made available by the authors, without undue reservation.
