# Supplementary material for: Prognostic value of the video head impulse test in sudden sensorineural hearing loss with vertigo: a systematic review and meta-analysis
Source: Front Neurol. 2026 Jan 12;16:1756795. doi: 10.3389/fneur.2025.1756795 (PMC12832497; doi:10.3389/fneur.2025.1756795)
Supplement: Supplementary file 1 [file Table_1.docx]

Table S1 Full search terms for each database

| **Database** | **Website** | **Search Strategys** | **Total** |
| --- | --- | --- | --- |
| Pubmed | http://www.ncbi.nlm.nih.gov/pubmed | ((("Hearing Loss, Sensorineural"[Mesh]) OR ((((((((((((((Hearing Loss,Sensorineural[Title/Abstract]) OR (Neurosensory Deafness[Title/Abstract])) OR (Deafnesses, Neurosensory[Title/Abstract])) OR (Neurosensory Deafnesses[Title/Abstract])) OR (Sensoryneural Deafness[Title/Abstract])) OR (Deafnesses, Sensoryneural[Title/Abstract])) OR (Sensoryneural Deafnesses[Title/Abstract])) OR (Deafness, Sensoryneural[Title/Abstract])) OR (Sensoryneural Deafness[Title/Abstract])) OR (Deafness, Neurosensory[Title/Abstract])) OR (Deafness Neurosensory[Title/Abstract])) OR (Sensorineural Hearing Loss[Title/Abstract])) OR (Hearing Loss, Cochlear[Title/Abstract])) OR (Cochlear Hearing Loss[Title/Abstract]))) AND (("Vestibular Function Tests"[Mesh]) OR (((((((vestibular function[Title/Abstract]) OR (vestibular function tests[Title/Abstract])) OR (Function Tests, Vestibular[Title/Abstract])) OR (Function Test, Vestibular[Title/Abstract])) OR (Tests, Vestibular Function[Title/Abstract])) OR (Test, Vestibular Function[Title/Abstract])) OR (Vestibular Function Test[Title/Abstract])))) AND (("Prognosis"[Mesh]) OR ((((((prognosis[Title/Abstract]) OR (Prognoses[Title/Abstract])) OR (Prognostic Factors[Title/Abstract])) OR (Prognostic Factor[Title/Abstract])) OR (Factor, Prognostic[Title/Abstract])) OR (Factors, Prognostic[Title/Abstract]))) | 102 |
| Embase | www.embase.com | ('perception deafness'/exp )OR ('hearing loss,sensorineural':ab,ti OR 'neurosensory deafness':ab,ti OR 'deafnesses, neurosensory':ab,ti OR 'neurosensory deafnesses':ab,ti OR 'deafnesses, sensoryneural':ab,ti OR 'sensoryneural deafnesses':ab,ti OR 'deafness, sensoryneural':ab,ti OR 'sensoryneural deafness':ab,ti OR 'deafness, neurosensory':ab,ti OR 'deafness neurosensory':ab,ti OR 'sensorineural hearing loss':ab,ti OR 'hearing loss, cochlear':ab,ti OR 'cochlear hearing loss':ab,ti OR 'perception deafness':ab,ti ) AND ('video head impulse test') | 140 |
| Scopus | www.scopus.com | (TITLE-ABS-KEY ( "Hearing Loss,Sensorineural" OR "Neurosensory Deafness" OR "Deafnesses, Neurosensory" OR "Neurosensory Deafnesses" OR "Sensoryneural Deafness" OR "Deafnesses, Sensoryneural" OR "Sensoryneural Deafnesses" OR "Deafness, Sensoryneural" OR "Sensoryneural Deafness" OR "Deafness, Neurosensory" OR "Deafness Neurosensory" OR "Sensorineural Hearing Loss" OR "Hearing Loss, Cochlear" OR "Cochlear Hearing Loss" ) AND TITLE-ABS-KEY ( "video head impulse test" ) ) | 117 |
| Cochrane Library | www.cochranelibrary.com | (Hearing Loss,Sensorineural):ti,ab,kw OR (Neurosensory Deafness):ti,ab,kw OR (Deafnesses, Neurosensory):ti,ab,kw OR (Neurosensory Deafnesses):ti,ab,kw OR (Sensoryneural Deafness):ti,ab,kw OR (Deafnesses, Sensoryneural):ti,ab,kw OR (Sensoryneural Deafnesses):ti,ab,kw OR (Deafness, Sensoryneural):ti,ab,kw OR (Sensoryneural Deafness):ti,ab,kw OR (Deafness, Neurosensory):ti,ab,kw OR (Deafness Neurosensory):ti,ab,kw OR (Sensorineural Hearing Loss):ti,ab,kw OR (Hearing Loss, Cochlear):ti,ab,kw OR (Cochlear Hearing Loss):ti,ab,kw AND (video head impulse test):ti,ab,kw | 4 |
