# Supplementary material for: Prognostic value of the video head impulse test in sudden sensorineural hearing loss with vertigo: a systematic review and meta-analysis
Source: Front Neurol. 2026 Jan 12;16:1756795. doi: 10.3389/fneur.2025.1756795 (PMC12832497; doi:10.3389/fneur.2025.1756795)
Supplement: Supplementary file 2 [file Table_2.docx]

Table S2 Risk of bias assessments according to the Newcastle-Ottawa Scale

| **References** | **Selection** | | | | **Comparability** | **Exposure/Outcome** | | | **Total** |
| --- | --- | --- | --- | --- | --- | --- | --- | --- | --- |
| Liu Y (2025) | ★ | ☆ | ★ | ★ | ☆ | ★ | ☆ | ☆ | 4 |
| Qian Y (2024) | ★ | ★ | ★ | ★ | ★ | ★ | ☆ | ☆ | 6 |
| Nakamichi N (2024) | ★ | ☆ | ★ | ★ | ☆ | ★ | ☆ | ☆ | 4 |
| Liu Y (2023) | ★ | ☆ | ★ | ★ | ☆ | ★ | ☆ | ☆ | 4 |
| Hong JP (2023) | ★ | ★ | ★ | ★ | ★ | ★ | ☆ | ☆ | 6 |
| Hong JP (2023) | ★ | ☆ | ★ | ★ | ☆ | ★ | ☆ | ☆ | 4 |
| Hao W (2023) | ★ | ☆ | ★ | ★ | ☆ | ★ | ★ | ☆ | 5 |
| Cho JW (2023) | ★ | ☆ | ★ | ★ | ☆ | ★ | ☆ | ☆ | 4 |
| Seo HW (2022) | ★ | ★ | ★ | ★ | ★ | ★ | ☆ | ☆ | 6 |
| Jiang Z (2021) | ★ | ★ | ★ | ★ | ★ | ★ | ☆ | ☆ | 6 |
| Lee JY (2020) | ★ | ☆ | ★ | ★ | ☆ | ★ | ☆ | ☆ | 4 |
| Byun H (2020) | ★ | ★ | ★ | ★ | ★ | ★ | ☆ | ☆ | 6 |
| Pogson JM (2016) | ★ | ☆ | ★ | ★ | ☆ | ★ | ☆ | ☆ | 4 |

The black stars represent the scores obtained, while the white stars represent the scores not obtained. The total score is 9 points, with a score greater than 6 indicating high-quality research
